# Complexity and multi-factoriality of *Trypanosoma cruzi* sylvatic cycle in coatis, *Nasua nasua* (Procyonidae), and triatomine bugs in the Brazilian Pantanal

**DOI:** 10.1186/s13071-016-1649-4

**Published:** 2016-07-01

**Authors:** Fernanda Moreira Alves, Juliane Saab de Lima, Fabiana Lopes Rocha, Heitor Miraglia Herrera, Guilherme de Miranda Mourão, Ana Maria Jansen

**Affiliations:** Laboratório de Biologia de Tripanossomatídeos, Fundação Oswaldo Cruz, Rio de Janeiro, Rio de Janeiro, Brasil; Laboratório de Vida Selvagem, Empresa Brasileira de Pesquisa Agropecuária (Embrapa)/Pantanal, Corumbá, Mato Grosso do Sul Brasil; Programa de Pós-Graduação em Ecologia e Monitoramento Ambiental, Universidade Federal da Paraíba–Campus Litoral, Rio Tinto, Brasil; Laboratório de Parasitologia Animal, Universidade Católica Dom Bosco, Campo Grande, Mato Grosso do Sul Brasil

**Keywords:** *Trypanosoma cruzi*, *Nasua nasua*, *Rhodnius*, *Triatoma*, Pantanal, Sylvatic cycle

## Abstract

**Background:**

*Trypanosoma cruzi* is dispersed in nature through many transmission mechanisms among a high diversity of vectors and mammalian species, representing particular behaviors and habitats. Thus, each locality has a unique set of conditions underlying the maintenance of this parasite in the wild. The aim of the present study was to evaluate the life-cycle of *T. cruzi* in free-ranging coatis from the central region of the Brazilian Pantanal using a multi-factorial approach.

**Methods:**

Three methodological blocks were used in the present study: (i) We evaluated *T. cruzi* infection using serological (ELISA) and parasitological (hemoculture) tests in free-ranging coatis captured from October 2010 to March 2012. In addition, we characterized *T. cruzi* isolates as DTUs (Discrete Typing Units); (ii) We evaluated *Trypanosoma* infection in species of *Triatoma* and *Rhodnius* inhabiting coati arboreal nests from May to September 2012 using parasitological and molecular assays; and (iii) We analyzed a set of longitudinal data (from 2005 to 2012) concerning the effects of *T. cruzi* infection on this coati population. Herein, we investigated whether seasonality, host sex, and host age influence *T. cruzi* prevalence and patterns of infection.

**Results:**

The 2010–2012 period presented high seroprevalence on coatis (72.0 % ELISA) and a high percentage of individuals with infectivity competence (20.5 % positive hemoculture). All isolates presented TcI band patterns, occurring in single (*n* = 3) and mixed infections (1 TcI/*T. rangeli*; 4 with undefined characterization). Both male and female individuals presented the same transmission potential, expressed as positive hemoculture, which was only detected in the summer. However, overall, the data (2005–2012) highlighted the importance of females for *T. cruzi* maintenance in the winter. Moreover, twenty-three (67.6 %) bugs from five coati nests (71.4 %) were infected with flagellated forms. Seventeen samples were characterized as *T. cruzi* (TcI and TcIII genotypes).

**Conclusion:**

In the Pantanal region, *T. cruzi* is transmitted in a complex, multifactorial, dynamic and non-linear transmission web. The coati nests might be inserted in this web, acting as important transmission foci at the arboreal stratum to different mammal species with arboreal or scansorial behavior.

## Background

Most scientific knowledge is based on simplification analyses, including reductionist methodologies and linear and deterministic concepts. Both reductionism and determinism are closely related. The former relies on the analysis of a system through its individual components, while the latter supports the idea that every phenomenon in nature is determined by pre-existing causes, and each cause produces a unique effect (and vice versa), sustaining the concept of predictability [1]. However, many biological systems, including parasitism, are complex systems [2]. Complex systems exist on the edge of chaos: they might present regular and predictable behaviors, but can also show nonlinear conduct (unpredictability) in response to minor modifications [1]. Thus, in parasitology, one must consider the likelihood of unpredictable modifications.

*Trypanosoma cruzi* is a hemoflagellate parasite and the causative agent of Chagas disease in humans [3]. This species represents a heterogeneous group, partially explained by hybridization events [4, 5]. Currently, the international consensus defines six discrete typing units (DTUs) within this species: TcI-VI [6]. In nature, this protozoan is maintained through distinct and complex cycles via several transmission mechanisms among a wide range of vectors and mammalian host species across almost all American habitats [7, 8]. The vector bugs (Reduviidae: Triatominae) become infected through the ingestion of mammalian blood at the parasitemic phase of infection. Thus, infective competence of a mammalian host depends on high parasitemia. Under natural conditions, transmission to mammals occurs through (i) contact of the contaminated feces of the vector with mammalian mucosa or injured skin during the blood meal (contaminative route); (ii) predation of infected bugs and mammals or ingestion of contaminated foods and triatomine feces (oral route); and (iii) congenital route [8]. The ecology of *T. cruzi* is, therefore, immensely variable, and each locality has a unique set of conditions underlying the emergence and persistence of this parasite in wildlife. Therefore, many aspects of *T. cruzi* cycles are poorly understood. Furthermore, few studies have evaluated how host-vector interactions modulate the *T. cruzi* cycle at a given locality [9–12].

In the Nhecolândia region of the Brazilian Pantanal biome, *T. cruzi* infection in the brown-nosed coati, *Nasua nasua* (Procyonidae), has been extensively examined between 2005–2010. We previously observed the importance of these mammals as reservoirs of the main *T. cruzi* lineages, representing a transmission hub for *T. cruzi* dispersion. Both males and females and all age groups have been continuously exposed to the infection [13–15]. However, females were the principal potential dispersers of *T. cruzi*, presenting higher rates of parasitemia, evaluated using hemoculture, primarily during the winter season [14].

Coatis are gregarious, diurnal, scansorial, and generalist mammals [16], feeding mainly on arthropods and fruits [17]. They present a particular behavior of constructing arboreal nests for resting and birthing [18]. In the Nhecolândia region, these nests are constructed at different sites: open areas, along forest edges and within the forest [19]. Furthermore, coatis frequent different nests to rest on a daily basis. Additionally, these nests can be communal (JS de Lima, personal observation). Recent findings in this region have shown that a third of the sampled resting nests are infested with triatomine bugs belonging to the genera *Rhodnius* and *Triatoma*. Indeed, insects at different nymphal stages and adult specimens have been observed in these nests, indicating successful colonization. Precipitin tests revealed that these bugs fed on coati, bird, rodent and marsupial species [20]. Additionally, this habitat might act as a point of convergence and dispersion for triatomine bugs and mammal hosts infected with *T. cruzi* in the Pantanal region [21].

Herein, we continued the longitudinal study of *T. cruzi* transmission cycle in the Pantanal biome focusing on the DTU characterization of *T. cruzi* isolates from triatomine bugs inhabiting coati resting nests. We also describe *T. cruzi* infection in the same coati population, including an evaluation of previous data [13–15] and the results obtained in the present study, corresponding to a seven-year longitudinal period.

## Methods

### Study area

Fieldwork was conducted at the Nhumirim ranch (56°39′W, 18°59′S), situated in the central region of the Pantanal, municipality of Corumbá, Brazilian state of Mato Grosso do Sul. This region is characterized by a mosaic of semideciduous forest, arboreal savannas, seasonally flooded fields covered by grasslands with dispersed shrubs and several temporary and permanent ponds [22]. The Pantanal is the largest Neotropical floodplain and is known for its biodiversity. Two well-defined seasons are recognized: a rainy summer (October to March) and a dry winter (April to September) [23]. Additionally, this area is subjected to multi-annual cycles of high flood and severe drought years [24]. Seasonal flood-drought cycles are the most striking ecological phenomena of the Pantanal, resulting in drastic changes in the landscape [25, 26].

### Coati capture

Ten field expeditions were performed from October 2010 to March 2012 as a follow-up to the longitudinal studies conducted from March 2005 to February 2007 [13], May 2007 to February 2009 [14] and August 2009 to April 2010 [15]. Box traps, made of galvanized wire mesh, were baited with bacon. The captured animals were immobilized through intramuscular administration of tiletamine hydrochloride plus zolazepan hydrochloride (10 mg/kg) prior to ear-tag identification, blood sampling, weighing and sex identification. Based on dental conditions and body size measurements, coatis were classified as adult (> 2 year-old), subadult (between 6 month- and 2 year-old) or juvenile (< 6 month-old) [27]. Blood samples (5–10 ml) were obtained through the external saphenous vein puncture and stored in vacuum tubes (DB Vacutainer®, São Paulo, São Paulo, Brazil) containing EDTA for hemoculture and without anticoagulant for serum obtainment. The animals were released at the site of capture after full recovery from anesthesia. From October 2010 to March 2012, we obtained 40 samples from 35 individuals (22 males, 13 females; 30 adults, 1 subadult and 4 juveniles), resulting from 5 recaptures. The total capture effort included 1,188 trap nights.

### Assessment of *T. cruzi* infection in coatis and interpretation of the results

To evaluate *T. cruzi* infection in coatis, we performed parasitological and serological assays. Hemoculture (HC) was accomplished under sterile conditions through the inoculation of 0.3 ml of each blood sample in NNN (Novy-McNeal-Nicolle) medium with a Liver Infusion Tryptose (LIT) overlay. The tubes were examined bi-weekly for 5 months. In case of trypanosomatid detection, the parasites were grown in LIT until log phase, cryopreserved, and deposited in the *Trypanosoma* Collection of Wild and Domestic Mammals and Vectors-COLTRYP (Fiocruz, Rio de Janeiro, Brazil) under accession numbers: 358, 360, 366–369, 476 and 477.

Additionally, we performed Enzyme-Linked Immunosorbent Assay (ELISA) using EIE-Chagas-Bio-Manguinhos kits (Bio-Manguinhos, Fiocruz, Rio de Janeiro, Rio de Janeiro, Brazil) kindly provided from the Laboratory of Diagnostic Technology/Fiocruz [28]. The cut-off value for the ELISA was defined as the mean optical absorbance of the negative controls plus 5 %. The anti-raccoon conjugate (Bethyl Laboratories, Inc., Montgomery, Texas, United States) was diluted 1:70,000, and each microtiter polystyrene plate contained 2 positive and 2 negative control samples.

Positive serological results indicate exposure to *T. cruzi* infection, while positive HC reveals significant *T. cruzi* parasitemia and thus a high potential for parasite transmission to triatomines. Seropositive individuals presenting negative results in the parasitological assay (HC) demonstrate sub-patent infection at the time of blood sampling.

### Parasitological analysis of triatomine bugs

The capture and identification of triatomine bugs is described elsewhere [20]. Triatomine specimens were stored live in Falcon® tubes and submitted for *Trypanosoma* infection diagnosis to the Laboratory of Trypanosomatid Biology at Fiocruz, Rio de Janeiro, Brazil (transfer time: 24–48 h; mortality: 10.8 %).

We evaluated *Trypanosoma* spp. infection in 34 triatomine bugs from seven coati resting nests collected monthly from May to September 2012: 13 adults (11 *Triatoma sordida* and two *Rhodnius stali*) and 21 nymphs (five *Triatoma* sp., 13 *Rhodnius* sp. as well as three non-identified nymphs). Because of the lack of genital development, the nymphs were classified at the genus level.

Parasitological analysis was performed through fresh examination of the gut content macerated in 0.85 % saline solution supplemented with 10 % antimycotic/antibiotic solution (A5955, Sigma®, São Paulo, Brazil). The samples were examined under a light microscope for the presence of flagellated forms. The positive samples were further subjected to: (i) cultivation in NNN/LIT medium for posterior DNA extraction of the successfully isolated parasites (COLTRYP accession numbers: 483, 485, 491,492, 497–500, 507–512, 515 and 517); and (ii) direct DNA extraction using the Gentra® Puregene® kit (Qiagen®, Gaithersburg, Maryland, United States) according to the manufacturer’s instructions.

### DNA extraction and molecular characterization

The isolates were washed in phosphate-buffered saline (0.15 M, pH = 7.2; centrifugation method: 4,000 rpm for 15 min at 4 °C). After the third procedure, the pellets were stored at -20 °C overnight. The pellets were resuspended in 50 μl of TE solution (10 mM Tris and 1 mM EDTA, pH 8.0) and incubated (56 °C for 2 h) with 10 μl of proteinase K (5 mg/ml) and 50 μl of 10 % sodium dodecyl sulfate. The genomic DNA was extracted three times with 500 μl of phenol-chloroform (1:1) and once with 500 μl of chloroform prior to precipitation with 45 μl of 3 M sodium acetate and 900 μl of ethanol [29]. The pellets obtained after centrifugation were suspended in 100 μl of distilled water. The DNA concentration was estimated after measuring the absorbance at 260 nm. The final template concentration (50 ng/μl) was achieved after dilution in distilled water.

Multiplex PCR amplification of the non-transcribed spacer of the mini-exon gene was performed [30]. This target classifies the *T. cruzi* population as TCI (corresponding to TcI DTU), TCII (TcII, TcV or TcVI DTUs) and zymodeme 3 (TcIII or TcIV DTUs) [31], and also detects *Trypanosoma rangeli*. Samples presenting TcII/TcV/TcVI or TcIII/TcIV band patterns were subjected to PCR amplification of the two targets: (i) the nuclear 1f8 gene, followed by restriction fragment length polymorphism (RFLP) analysis of DNA fragments digested with Alw21I enzyme [32], and/or (ii) histone 3 (H3), followed by RFLP analysis of the DNA fragments digested with Alul enzyme [33].

All reactions included sterile distilled water as a negative control and samples from *T. cruzi* strains of each genotype as positive controls. After electrophoresis, the amplified PCR products were visualized in ethidium bromide-stained agarose gels (2 %) under ultraviolet light.

### Longitudinal data of *T. cruzi* infection in coatis from March 2005 to March 2012

We assembled all data associated with *T. cruzi* infection in coatis from previously published studies [13–15] and the present study, corresponding to a seven-year longitudinal period (March 2005 to March 2012). The data comprised 220 individuals. Sixty-one individuals were captured from two to six times, totaling 317 captures (Table 1).

**Table 1:** Sampled coatis, captured from March 2005 to March 2012 at the Brazilian Pantanal

Season	Male		Female		Total captured
	S–SA–J	Total	S–SA–J	Total	
Summer	101–31–10	142 (67 %)	61–8–5	74 (70 %)	216
Winter	54–14–1	69 (33 %)	26–3–3	32 (30 %)	101
Total	155–45–11	211	87–11–8	106	317

*Abbreviations*: A, adult; SA, subadult, J, juvenile

We performed ELISA on all coati sera samples to compare the results with those of previous studies using an indirect immunofluorescence assay.

### Statistical analysis

To analyze *T. cruzi* exposure in coatis from October 2010 to March 2012, we investigated differences between seroprevalence and positive HC rates for coati gender (Fisher’s exact test, α = 0.05). We did not evaluate the influence of age and seasonality, reflecting the small amount of records obtained during this period.

For the 2005–2012 period, we investigated differences in seroprevalence and positive HC rates between coati genders (Chi-square test, α = 0.05). Depending on load samples, both Fisher’s exact and Chi-square tests were used to access potential differences between positive HC rates for coati sex clustered according to age.

## Results

### *Trypanosoma cruzi* in coatis from October 2010 to March 2012

The results corroborate the maintenance of the essential role of the coati population in *T. cruzi* sylvatic cycle, as demonstrated by the high seroprevalence (72.0 % positive ELISA, *n* = 29) and high percentage of individuals with infectivity competence observed throughout the study period (20.5 % positive HC, *n* = 39) (Table 2). These rates did not differ among male and female individuals [Fisher’s exact test: ELISA (*P* > 0.99, *n* = 29); HC (*P* = 0.40, *n* = 39)]. Positive HC results were only detected in the summer (January - February-March). In addition, 21.7 % seropositive individuals exhibited significant parasitemia (i.e. positive HC; *n* = 23).

**Table 2:** Seroprevalence (ELISA) and hemoculture (HC) of *Trypanosoma cruzi* in coatis from October 2010 to March 2012

October 2010 - March 2012	Positive ELISA			Positive HC		
	Male	Female	Total	Male	Female	Total
	% (*n*)	% (*n*)	% (*n*)	% (*n*)	% (*n*)	% (*n*)
Adult	81.2 (16)	77.8 (9)	80 (25)	17.4^a^ (23)	27.3^b^ (11)	20.6 (34)
Subadult	0 (1)	–	0 (1)	0 (1)	–	0 (1)
Juvenile	50 (2)	0 (1)	33.3 (3)	0 (2)	50 (2)	25 (4)
Total	73.7 (19)	70 (10)	72 (29)	15.4 (26)	30.8 (13)	20 (39)

*n* = total number of samples analyzed

^a^Four samples from four recaptured male coatis

^b^One sample from one recaptured female coati

Coatis were exposed to *T. cruzi* infection early in life, demonstrated as positive HC and ELISA results, respectively, observed in male and female individuals younger than six months.

Molecular characterization showed that all *T. cruzi* isolates (*n* = 8) presented TcI band patterns in single (samples 4, 6 and 7) and mixed infections with *T. rangeli* (sample 5). Furthermore, *Trypanosoma rangeli* occurred in three individuals (numbers 3, 5, and 8) from the 39 evaluated samples (7.7 %).

**Fig. 1:**
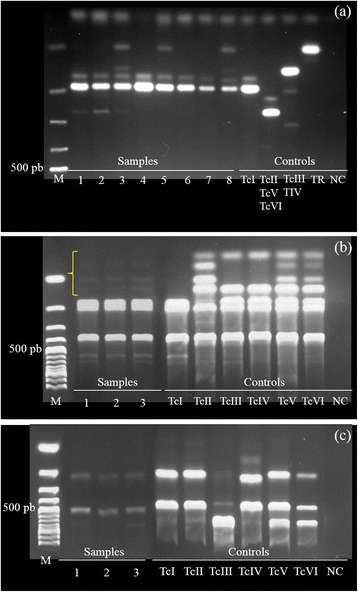
*Trypanosoma cruzi* genotyping from coatis at in the Pantanal region, Brazil. Agarose electrophoresis gels of (**a**) mini-exon multiplex PCR products; (**b**) 1f8 gene/Alw21I PCR-RFLP products; and (**c**) H3/Alul PCR-RFLP products. The brace in 1b shows non-characterizable weak bands. *Abbreviations*: M, Molecular weight markers (100 bp DNA ladder); TR, *T. rangeli*; NC, Negative control

We could not define the genotype of samples 1, 2, 3 and 8, because of the lack of correspondence between the results of the mini-exon gene PCR (Fig. 1a) and the results of 1f8/Alw21I and H3/Alul assays (Fig. 1b, c). For example, the results of the mini-exon gene PCR of samples 1 and 2 displayed mixed infection with TcI + TCII (Fig. 1a), while 1f8/Alw21I and H3/Alul assays displayed only infection with TcI (Fig. 1b, c). Additionally, sample 3 presented a multiple band pattern in the multiplex PCR (Fig. 1).

### *Trypanosoma cruzi* infection in coatis from March 2005 to March 2012

**Fig. 2:**
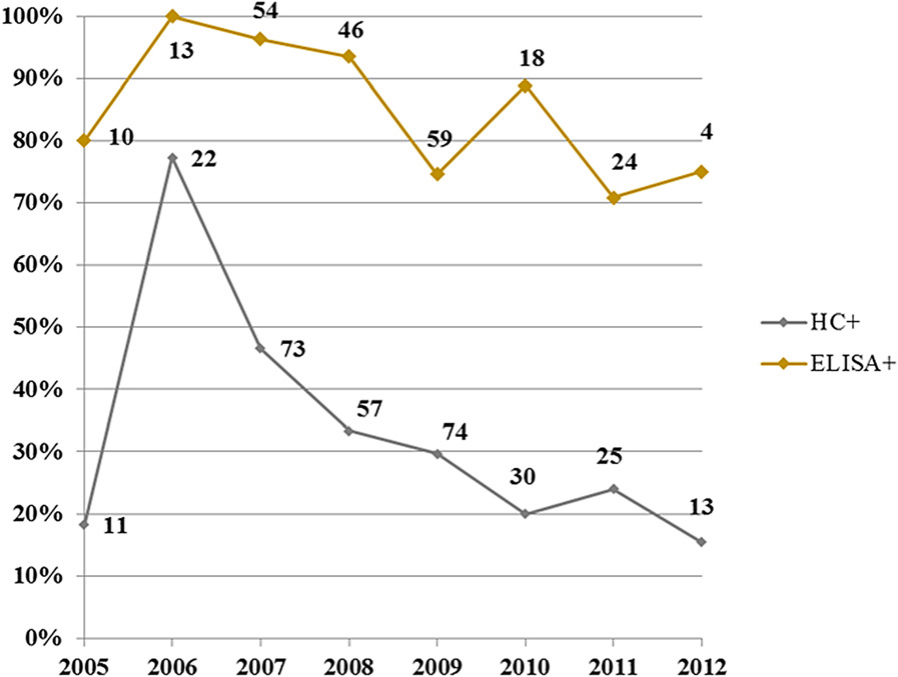
Annual fluctuations of positive HC and ELISA rates of *T. cruzi* infection in coatis from the Brazilian Pantanal. The numbers close to the dots correspond to the sample size

The sylvatic cycle of *T. cruzi* was highly stable, demonstrated as high annual proportions of seropositive coatis. Moreover, the role of the coati population as a *T. cruzi* reservoir was maintained during the entire period, as a relatively large portion of the population presented positive HC results each year (Fig. 2).

**Fig. 3:**
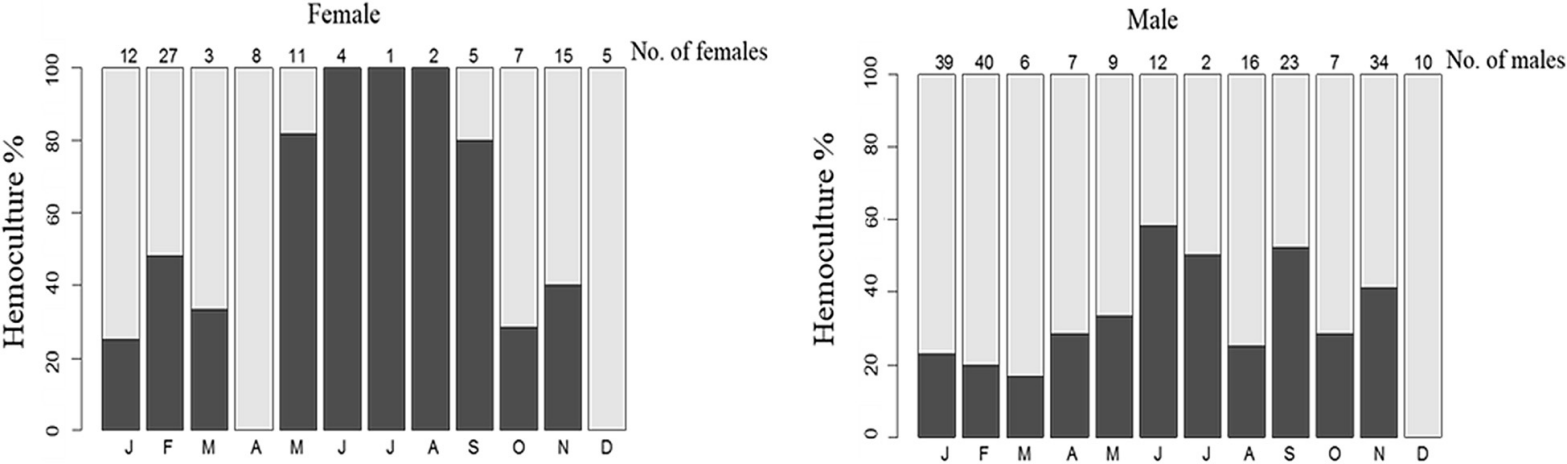
Proportion of female and male coatis with transmission potential (positive hemoculture) per month (accumulated from 2005 to 2012). The number of sampled coatis is indicated above the bars

Although positive HC results were only detected in the summer during October 2010 to March 2012, all seven-year data showed that both male and female coatis presented higher HC rates in the winter. Moreover, this pattern was highly expressed in females (Fig. 3). The importance of females in *T. cruzi* transmission is summarized in Table 3.

**Table 3:** Seroprevalence (ELISA) and hemoculture (HC) of *Trypanosoma cruzi* in coatis from March 2005 to March 2012

2005 - 2012	Positive HC			Positive ELISA		
	Male	Female	*P*-value	Male	Female	*P*-value
	% (*n*)	% (*n*)		% (*n*)	% (*n*)	
Adult	30.7 (150)	42.7 (82)	*P* = 0.07; χ^2^ = 3.369	85.9 (92)	93.8 (48)	*P* = 0.27; χ^2^ = 1.235
Subadult	32.5 (43)	63.6 (11)	*P* = 0.08; Fisher’s exact test	79.2 (24)	85.7 (7)	*P* = 0.64; Fisher’s exact test
Juvenile	27.3 (11)	37.5 (8)	*P* > 0.99; Fisher’s exact test	88.9 (9)	83.3 (6)	*P* > 0.99; Fisher’s exact test
Total	30.9 (204)	44.5 (101)	*P* = 0.03^a^; χ^2^ = 5.521	84.8 (125)	91.8 (61)	*P* = 0.27; χ^2^ = 1.220 with Yates correction

*n* = total number of samples analyzed

^a^Statistical significance at 95 % confidence

### *Trypanosoma cruzi* infection in triatomine bugs

Twenty-three (67.6 %; *n* = 34) triatomine bugs (*Triatoma* sp.: 56.2 %, *n* = 16; *Rhodnius* sp.: 80.0 %, *n* = 15) from five coati nests (71.4 %; *n* = 7) were infected with flagellated forms (positive fresh exam) [20]. Among the five coati nests harboring infected bugs, the infection rate in each nest was high, varying from 66.6 to 100 %.

**Fig. 4:**
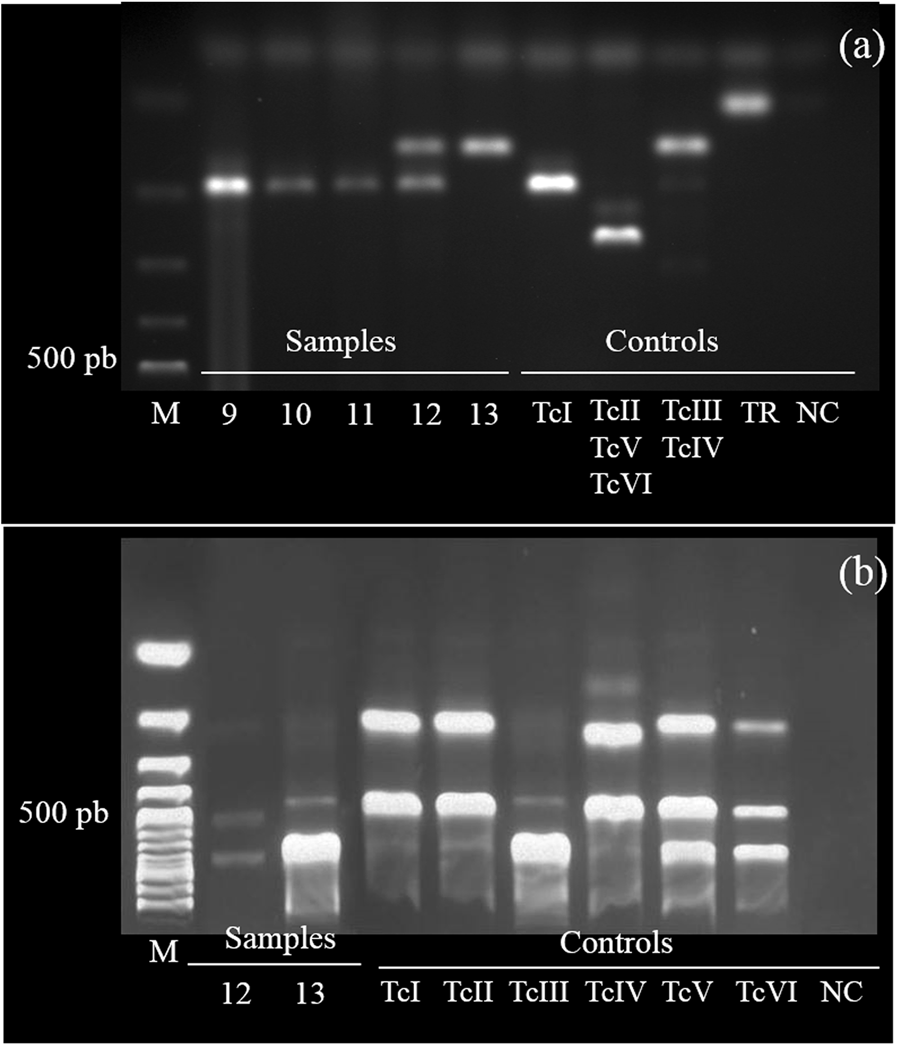
*Trypanosoma cruzi* genotyping from triatomine bugs in the Pantanal region, Brazil. Agarose electrophoresis gels of (**a**) mini-exon multiplex PCR products (representative samples); and (**b**) H3/Alul PCR-RFLP products. *Abbreviations*: M, Molecular weight markers (100 bp DNA ladder); TC, *T. cruzi*; TR, *T. rangeli*; NC, Negative control

Seventeen samples of four coati nests were initially characterized based on a mini-exon gene. All specimens were infected with *T. cruzi*: 13 single infections with TcI (76.4 %), a single infection with Z3 (5.8 %), and three co-infections with TcI and Z3 (17.6 %) (Fig. 4). PCR-RFLP analysis of the H3 target in two samples showed that Z3 corresponded to the TcIII DTU (Fig. 4). Two samples (TcI/Z3) could not be genotyped to DTUs. The TcI genotype was the most widely distributed, occurring in all sampled nests. On the other hand, Z3 was restricted to two nests and in *Triatoma* sp. bugs (Table 4).

**Table 4:** *Trypanosoma cruzi* subpopulations in triatomine bugs per coati nest

Nest	Species	Sampling method	
		Gut content culture	Direct DNA extraction
A	*Rhodnius stali*	TcI	na
A	*Rhodnius* sp.	TcI	na
A	*Rhodnius* sp.	TcI	na
A	*Rhodnius* sp.	TcI	na
A	*Rhodnius* sp.	TcI	TcI
A	*Rhodnius* sp.	TcI	na
A	*Rhodnius* sp.	Nd	na
A	*Triatoma sordida*	TcI	TcI
A	*Triatoma sordida*	TcI	na
A	*Triatoma sordida*	TcI	na
A	*Triatoma sordida*	Nd	na
A	*Triatoma sordida*	TcI	TcI/Z3
A	*Triatoma* sp.	TcIII	Z3
A	*Triatoma* sp.	TcI	na
A	Unidentified	Nd	na
B	*Rhodnius stali*	TcI	nd
B	*Rhodnius* sp.	TcI	nd
C	*Rhodnius* sp.	TcI	na
C	*Rhodnius* sp.	Nd	na
D	*Triatoma sordida*	Nd	TcI/Z3
D	Unidentified	TcI/TcIII	nd
E	*Triatoma sordida*	Nd	na

*Abbreviations*: *nd*, not done; *na*, no amplification

One sample presented two different results concerning the *T. cruzi* subpopulation: the gut content culture showed single infection by TcI, whereas the direct DNA extraction of the gut content revealed mixed infection with TcI and Z3 (DTU characterization was not performed) (Table 4).

## Discussion

The importance of the coati as a *T. cruzi* reservoir in the Nhecolândia region has been well recognized [13–15]. Herein, we confirmed the potential role of this population, particularly female individuals in the winter, in maintaining high and stable parasitemia, as observed in all data obtained during the entire seven-year period. Interestingly, the data obtained from October 2010 to March 2012 did not show this winter pattern, suggesting the unpredictability of the *T. cruzi* cycle and the role of a mammal host as a reservoir in a given area.

Indeed, in the two tours of Rocha FL at Nhumirim ranch from August 20 to August 31, 2013 (capture effort: 165 trap nights) and from August 18 to August 28, 2014 (capture effort: 180 trap nights), a distinct enzootic frame was revealed: low relative abundance of coatis (six and two captured coatis in 2013 and 2014, respectively) and undetected positive hemoculture (*n* = 8; unpublished results). Thus, the occurrence of a host population with a high transmission potential and the relative abundance and contact rate of the mammalian and vector populations are crucial factors for the epizootiology of *T. cruzi* in a given area [34]. These results suggest that the Pantanal climate might have changed coati abundance. The severe drought period in 2013 likely killed many animals and forced the emigration of resistant individuals. These findings show the complexity and unpredictability of *T. cruzi* transmission and highlight the influence of abiotic factors on host-parasite dynamics [35].

One feature of complex systems is the synergy of the components, thus, scientific methodology cannot reduce or deduce a system from the simplest parts [1]. Thus, the study of the *T. cruzi* cycle in a single species, the coati, represents a facet of the manifold host-parasite interactions occurring in the study region. Therefore, we further extended these studies to the role of *T. cruzi* vectors.

Triatomine bugs living in close association with free-ranging coatis have been observed in the Nhecolândia region [20, 21]. Herein, we observed that the majority of these nests sheltered triatomine bugs infected with *T. cruzi*, suggesting that coatis might be infected early in these nests through two mechanisms, the oral route and the contaminative route.

Furthermore, the coati nests might act as transmission foci in the arboreal stratum, where different *T. cruzi* populations are dispersed among vectors and different mammal species with arboreal or scansorial behaviors. Triatomine bugs can present habitat restriction and relatively low mobility when living in close association with a mammal species [36, 37], particularly in well-fed populations [38, 39]. However, a nest can be visited by different coati individuals and other mammalian species, including marsupials and rodents, as revealed through precipitin tests of triatomine feces and a camera trap record of a spiny rat (*Thrichomys fosteri*) visiting an abandoned coati nest [20]. The dynamics inside these shelters enhances the likelihood of *T. cruzi* dispersion.

We observed *T. rangeli* infection rate of 7.7 % in the coati population. Natural infections with *T. rangeli* in vectors have been reported in Brazil [40, 41], primarily in the Amazon region [42, 43]. However, this species was not detected in any of the 34 sampled bugs (Table 4), suggesting a niche for *T. rangeli* transmission other than the triatomine species inhabiting coati nests.

We observed the selective forces of axenic culture medium and highlighted the importance of DNA extraction directly from the gut content (Table 4). Parasite growth in vitro is an additional intermediate step that might have selected the TcI population to the detriment of Z3, resulting in a non-representative isolate [44]. This issue must be always considered when studying cultured isolates [45, 46]. Therefore, the detection of a particular subpopulation through growth in culture medium does not exclude mixed infections.

We could not define the genotype of *T. cruzi* isolates from four coatis, as the mini-exon band patterns were not consistent with the results of the RFLP analysis (Fig. 1). Additionally, we observed an isolate (sample 3) presenting multiple bands in the mini-exon target, as previously reported in four coati individuals [15]. These misinterpretations reflect heterogeneous populations with high genetic diversity isolated from free-ranging sylvatic hosts [47, 48], particularly with respect to coatis. This species displays many biological traits (eclectic feeding behavior, long-lived, long distance dispersal and both arboreal and terrestrial strata exploration in different habitats) that enhance the likelihood of exposure to various and heterogeneous *T. cruzi* subpopulations.

## Conclusions

The essential role of coatis in *T. cruzi* sylvatic cycle was maintained during the 2010–2012 period. However, many biotic and abiotic factors might change the pattern of *T. cruzi* infection in a given population and the epizootiological profile. Therefore, in the Pantanal region, *T. cruzi* is transmitted in a complex, multifactorial, dynamic and non-linear transmission web. Coati nests might be inserted in this web, acting as important transmission foci at the arboreal stratum to different mammal species with arboreal or scansorial behaviors.

### Ethics statement

This study was approved through the Ethical Commission for Experimentation with Animal Models (CEUA) of Fiocruz (registration number: P-292-06). The capture and sample collection were performed according to the Brazilian Government Institute for Wildlife and Natural Resources Care (IBAMA) regulations (license numbers 25078-2/2010, 28772-1/2011, 38787-1/2013, 38787-2/2014). Appropriate biosecurity techniques and individual protection equipment were used in all procedures for the collection and handling of the biological samples.
